# Antileishmanial, Antimicrobial and Antifungal Activities of Some New Aryl Azomethines

**DOI:** 10.3390/molecules15020660

**Published:** 2010-01-28

**Authors:** Yasser M.S.A. Al-Kahraman, Hassan. M.F. Madkour, Dildar Ali, Masoom Yasinzai

**Affiliations:** 1 Institute of Biochemistry, University of Balochistan, Quetta, Pakistan; 2 Synthetic Organic Chemistry Lab., Chemistry Department, Faculty of Science, Ain Shams University, Cairo, Egypt

**Keywords:** azomethines, primary aromatic amines, hetaryl carboxyladehyes, antileishmanial activity, antifungal activity, antibacterial activity

## Abstract

A series of eighteen azomethines has been synthesized by the reaction of appropriate primary aromatic amines with aryl and/or heteroaryl carboxaldehydes. The synthesized azomethines have been evaluated for their *in vitro* antileishmanial, antibacterial and antifungal activities. The results revealed some antifungal activity of most of the synthesized compounds, whereas the antileishmaniasis activity results highlighted that all synthesized azomethines inhibited parasite growth and most of them showed highly potent action towards *Leishmania major* promastigotes. No remarkable bactericidal activities were observed.

## Introduction

Although the last century has been characterized by a drastic lowering in the mortality caused by infectious diseases, some diseases still represent a dreadful menace to human health and therefore, for a more efficient control, require the steady development of new, more powerful and inexpensive drugs. Leischmaniasis, considered a serious public health concern in many countries, is one of these diseases. It is now endemic in many parts of Pakistan and Balochistan and the Upper Sindh are vulnerable to cutaneous leishmaniasis. The World Health Organization (WHO) has identified leishmaniasis as a major and increasing public health problem [1]. In spite of the social and economic importance of leishmaniasis as a tropical infectious disease, efforts directed towards the discovery of new drugs against it remain undeveloped [2,3]. Moreover, the drugs currently in use are expensive, require long-term treatment [4], display high liver and heart toxicities, develop clinical resistance after few weeks of treatment and currently contribute to increase leishmaniasis-AIDS co-infections in some countries [5,6]. Azomethines are characterized by the –CH=N- (imino group) which has special importance in elucidating the mechanism of transmination and racemization in biological system [7,8,9]. Azomethines have high potential chemical permutation possibilities and show diuretic [10], anticancer [11,12,13,14], antibacterial [15,16,17,18,19], and antifungal activities [20,21,22]. This class of compounds has also exhibited activity against a wide range of organisms and is known to have medicinal importance and is used in drug design [23,24,25]. It has been also reported that when some aldehydes were functionalized by condensation with various amines, the resulting azomethines had antiparasitic activities [26].

## Results and Discussion

The present work is a continuation of our ongoing programme devoted to the utilization of simple and readily obtainable materials in the synthesis of different biologically active compounds [27,28,29,30,31,32,33], aiming at the same time to seek a cure for the cutaneous antileishmaniasis facing our society in Pakistan by presenting some cheap azomethines that might be useful in antileishmanial and antifungal drug development.

**Scheme 1:**
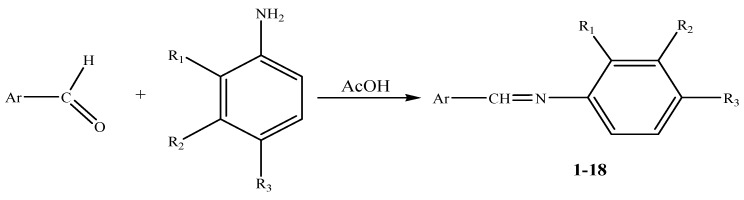

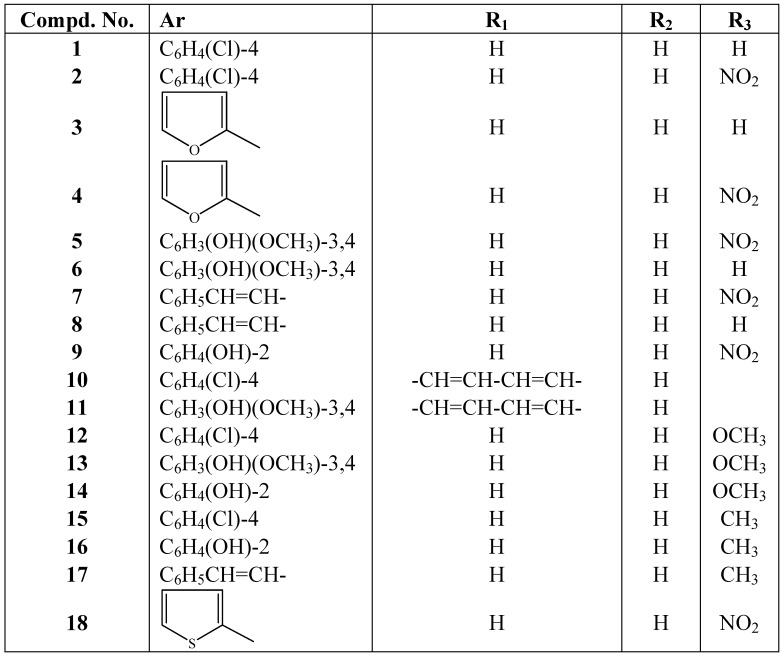
General synthetic route.

Reaction of various substituted anilines with aromatic and/or heterocyclic aldehydes in hot methanol containing a catalytic amount of glacial acetic acid afforded the target azomethines **1**-**18** in good yields (55–87%). (cf. Scheme 1).

The structural features of the synthesized azomethines were determined from the corresponding IR, ^1^H-NMR, ^13^C-NMR and mass spectroscopy data, listed in Table 1, Table 2, Table 3 and Table 4. All compounds showed in the IR spectra a strong absorption band at 1,592–1,637 cm^−1^, typical of the stretching vibrations of the C=N functionality, as well as the bands corresponding to C-H stretching vibrations.

The ^1^H-NMR spectra of compounds **1**-**18** contained multiple signals in the δ 6.90 to 8.08 ppm regions due to aromatic protons and a singlet at 8.43-9.75 ppm from the C-H protons of the CH=N groups. In the spectra of compounds **5**, **6**, **11**, **12**, **13** and **14**, signals between δ 3.50 and 4.0 ppm were attributed to –OCH_3_ groups. The signals at δ 2.36 and 2.33 ppm which appeared as singlets in compounds **15** and **16** were assigned for methyl groups. In the DEPT spectra of compounds **1**-**18**, there are no peaks at 189.0-196.0 ppm due to CHO group and the observed peaks were shifted to ~160.0-163.0 ppm which were attributable to HC=N group.

### Antileishmaniasis activity

Antileishmanial activity of the compounds (1-18) was assayed by Zhai’s method [40] using a pre-established culture of *L. major* (Table 5) Parasites were cultured in medium M199 with 10% foetal bovine serum; 25 mM of HEPES, and 0.22 µg of penicillin and streptomycin, respectively, at 24 °C in a shaking incubator. Compounds **1, 6** and **16** have moderate activity against *L major*. Azomethines **4, 10, 14, 15,** and **17** show good activity against *L major* and azomethines **9** and **12** show significant activity. The high *in vitro* antileishmaniasis activity of these compounds makes them promising leads for development of effective therapeutic agents.

### Antifungal activity

The antifungal bioassay is screened by the Agar Tube Dilution Method [41,42], in which the test compounds were screened for activity against the following organisms: *Candida albicans, Aspergillus flavus, Microsportum canis, Fusarium solani* and *Candida glabrata*. Table 6 shows % inhibition of the synthesized azomethines. In all tests the linear growth in control was 100 mm. It is evident from the antifungal screening that:

(i) None of the compounds are inactive against *C. albicans* or *C. glabrata*.
(ii) Only compound **17** has moderate activity against *A. flavus*.
(iii) Azomethines **4, 5, 12** and **14** have low activity against *M. canis*, whereas compound **9** has good antifungal activity against the same fungus.
(iv) Azomethine **9** shows moderate activity against *F. solani*, whereas azomethines **4, 11, 17** and **18** have low activity against the same fungus.

### Antibacterial activity

The antibacterial activity of the synthesized azomethines was screened by the agar well diffusion method [43,44] against Gram-negative and Gram-positive bacteria, namely *Escherchia coli*, *Bacillus subtilis, Shigella flexenari, Staphylococcus aureus, Pseudomonas aeruginosa* and *Salmonella typhi.* Only compound **4** showed significant activity (25 mm inhibition zone) against *E. coli* and moderate activity (14 mm zone) against *B. subtilis*.

## Experimental

### General

All aromatic and heterocyclic aldehydes as well as primary aromatic amines were obtained commercially from the Aldrich Chemical Company. Solvents used were of analytical grade. M.p. were measured using a Stuart (UK) electric melting point apparatus. IR spectra were recorded with a Thermo 330 FTIR spectrometer. Mass spectra were measured on a MAT 312 Finnigan instrument operating at 70 eV. ^1^H- and ^13^C-NMR spectra were recorded on a Avance 400 Bruker instrument using TMS as internal standard and DMSO-d_6_ as solvent.

### Chemistry: General procedure for synthesis of azomethines

An equimolar mixture of aryl and/or hetaryl carboxaldehyde with selected primary aryl amine in methyl alcohol (30 mL) containing few drops of glacial acetic acid was heated to reflux temperature for 3-4 hours then left overnight until the product precipitated. The product was filtered off, washed with light petroleum and recrystallized from the suitable solvent [cf. Table 1]. It is worthy to mention that anhydrous dry solvents for recrystallization were essential to avoid decomposition by hydrolysis. The crystalline solid that separated out was filtered by suction using water pump and washed with light petrol to afford the desired azomethines **1**-**18** in pure state.

### Biological Screening: Antileishmaniasis Activity - Preparation of Samples

Each compound (1 mg) was dissolved in DMSO (1 mL) and Amphotercin B (1 mg) was also dissolved in DMSO (1 mL) as positive control. Parasites at log phase were centrifuged at 3,000 rpm for 3 minutes. Parasites were diluted in fresh culture medium to a final density of 2 × 10^6^ cells/mL. In 96-well plates, 180 µL of medium was added in different wells. Twenty µL of the compound was added in medium and serially diluted. Parasite culture (100 µL) was added in all wells. Three rows were left for negative and positive controls. In the negative controls, DMSO was serially diluted in medium while the positive control contained varying concentrations of the standard antileishmanial compound Amphotercin B. The plates were incubated for 72 hours at 24 °C. The culture was examined microscopically on an improved neubaur counting chamber and IC_50_ values of compounds possessing antileishmanial activity were calculated. All assays were run in duplicate. IC_50_ of samples was determined by using the Prism software.

### Antifungal activity

The concentration of the tested compounds was 200 ug/mL of DMSO. A reference antifungal and DMSO were used as positive and negative control, respectively. Incubation temperature was 27 °C and time 7 days. The inculation of fungus was carried out with a 4 mm diameter piece of fungus removed from a seven-day-old culture. The growth in the compound-amended media was determined by measuring linear growth (mm) and growth inhibition calculated with reference to the negative control and calculating the % inhibition of fungal growth:

$$\%\;\mathrm{Inhibition}\text{=}100-\frac{\mathrm{linear}\;\mathrm{growth}\;\mathrm{in}\;\mathrm{test}\;(\mathrm{mm})}{\mathrm{linear}\;\mathrm{growth}\;\mathrm{in}\;\mathrm{control}\;(\mathrm{mm})}\times 100$$

### Antibacterial Activity

The concentration of tested compound was 1 mg/mL of DMSO, whereas the concentration of the reference antibacterial drug imipenem was 10 mg/disc. Blank test showed that DMSO in the preparations of the test solutions does not affect the test organisms.

## Conclusion

Eighteen azomethines were prepared by reacting an aromatic or heterocyclic aldehyde and an aromatic primary amine compound in a protic solvent like ethanol or methanol heated to reflux temperature in presence of few drops of acetic acid as a catalyst. The acidic catalyst leads to the increase of the electrophilicity of the caronyl function of the carboxalehyde containing reactant. The results revealed some order of fungicidal activities of most of the synthesized compounds whereas the results of leishmanicidal activity highlighted that all synthesized azomethines inhibited the growth of parasite and most of them showed high potent action towards leishmania major promastigotes. No remarkable bactericidal activities have been recorded. Azomethines are characterized by the –CH=N- (imino group) which has special importance in elucidating the mechanism of transmination and racemization in biological system. Azomethines have high potential chemical permutation and show diuretic, anticancer, antibacterial, and antifungal activities. This class of compounds also exhibited activity against a wide range of organisms and are known to have medicinal importance and used in drugs design. It has been also reported that some aldehydes when were functionalized by condensation with various amines, the produced azomethines had antiparasitic activities. It is evident that Azomethine compounds **1, 6** and **16** have moderate activity against *L major*. Azomethines **4, 10, 14, 15,** and **17** show good activity against *L major* and azomethine **9** and **12,** shows significant activity against *L.major*. The high *in vitro* Anti leishmaniasis activity of these compounds makes them a promising lead for development of effective therapeutic agents.It is clear that the synthesize of Azomethines showed only compound 17 has moderated activity against A flavus. Azomethine 4,5,12 and 14 have low activity against M. Canis whereas compound 9 has good anti fungal activity against same fungus. Azomethine 9 shows a moderate activity against F. Solani whereas azomehtines 4,11,17 and 18 have low activity against the same fungus. In bacteria among the 18 synthesize of azomethines tested only compound 4 showed significant activity against E.coli and low activity against B. subtilis.

## Figures and Tables

**Table 1:** Physical and select IR data of the synthesized azomethines **1**-**18**.

Compd.	Color	Molecular^a^ formula (Mol. Mass)	Mp (°C) (recryst. solvent)^b^	Yield (%)	IR (cm^−1^)		
					N=C	Azomethine C-H	Substituent
**1**	Pale yellow	C_13_H_10_ClN [34] (215.05)	72-4 (L.P)	82	1621	2874, 1352	­-
**2**	Yellow	C_13_H_9_ClN_2_O_2_ [35] (260.04)	140-42 (L.P)	74	1625	1333	1505, 1376(NO_2_)
**3**	Brown	C_11_H_9_NO [36] (171.07)	106 d (B)	79	1605	2850, 1328	3360(OH)
**4**	Brown	C_11_H_8_N_2_O_3_ [37] (216.05)	196-98 (EtOH)	68	1592	1285	1535, 1339(NO_2_)
**5**	Pale orange	C_14_H_12_N_2_OH (272.08)	148-50 (L.P)	64	1628	1325	3354–3480(OH) 1544, 1342 (NO_2_)
**6**	Pale yellow	C_14_H_13_NO_2_ (227.09)	155-57 (L.P)	72	1621	1388	3088 broad(OH)
**7**	Orange	C_15_H_12_N_2_O_2_ (252.09)	133d (MeOH)	65	1592	1300	1512, 1335(NO_2_)
**8**	Pale brown	C_15_H_13_N (207.10)	100-02 (MeOH)	66	1623	1375	-
**9**	Orange	C_13_H_10_N_2_O_3_ (242.07)	162-64 (L.P)	59	1632	1338	1562, 1347 (NO_2_), 3150 (OH)
**10**	Pale brown	C_17_H_12_ClN (265.07)	107-09 (MeOH)	78	1637	1391	-
**11**	Brown	C_18_H_15_NO_2_ (277.11)	114-16 (B)	76	1621	1381	3048(OH)
**12**	Pale Brown	C_14_H_12_ClNO (245.06)	136-37 (MeOH)	87	1629	2838, 1364	2838, 2895 (CH_3_)
**13**	Pale Brown	C_15_H_15_NO_3_ ( 257.11)	257-59 (MeOH)	70	1621	2954, 1378	3000 (broad OH), 2954, 2982 (CH_3_)
**14**	Greenish White	C_14_H_13_NO_2_ (227.09)	85-87 (MeOH)	67	1637	2964, 1362	3610(OH), 2964, 2996 (CH_3_)
**15**	White	C_14_H_12_ClN (229.07)	127-29 (MeOH)	79	1622	2929, 1354	2854, 2925 (CH_3_)
**16**	White	C_14_H_13_NO (211.10)	96-98 (MeOH)	67	1615	2920, 1366	3100 (OH), 2920 (CH_3_)
**17**	Pale Orange	C_16_H_15_N [38] (221.12)	76-77 (MeOH)	71	1627	2912, 1379	2912 (CH_3_)
**18**	Pale Yellow	C_11_H_8_N_2_O_2_S [39] (232.03)	135-37 (MeOH)	55	1612	1323	1543, 1341 (NO_2_)

^a^ references are given for known products, the others are new; ^b^ L.P = Light Petroleum b.p. 60–80 °C, B = benzene, EtOH = ethanol, MeOH = methanol.

**Table 2:** ^1^H-NMR of azomethines **5**-**16** (DMSO-d_6_) (δ, ppm).

Compd.	^1^H-NMR
**5**	9.45 (s, 1H, -OH, exchangeable), 8.36 (s, 1H, benzylidenimine H), 8.08-6.90 (m, 7H, Ar-H), 3.80 (s, 3H, -OCH_3_).
**6**	9.75 (s, 1H, -OH, exchangeable), 8.43 (s, 1H, -CH=N), 7.52-6.88 (m, 8H, Ar-H), 3.84 (s, 3H, -OCH_3_).
**7**	8.10 (m, 1H, -CH=N), 7.60-6.95 (m, 11H, Ar-H and olefinic H).
**8**	8.25 (d, 1H, benzylideimin H, *J* = 5.36 Hz), 7.54-7.07 (m, 12H, Ar-H and olefinic H).
**9**	12.27 (s, 1H, OH, exchangeable), 8.99 (s, 1H, -CH=N), 8.32-6.98 (m, 7H, Ar-H).
**10**	8.73 (s, 1H, benzylidenimine H), 8.09-7.23 (m, 11H, Ar-H).
**11**	9.76 (s, 1H, OH, exchangeable), 8.53 (s, 1H, benzylidenimine H), 8.29-6.92 (m, 10H, Ar-H), 3.89 (s, 3H, -OCH_3_).
**12**	8.64 (s, 1H, -CH=N), 7.93-6.97 (m, 8H, Ar-H), 3.77 (s, 3H, -OCH_3_).
**13**	9.60 (s, 1H, OH, exchangeable), 8.44 (s, 1H, -CH=N), 7.49-6.86 (m, 7H, Ar-H), 3.83 and 3.76 (2s, 6H, 2 -OCH_3_).
**14**	13.38 (s, 1H, OH, exchangeable), 8.59 (s, 1H, benzylidenimine H), 7.36-6.89 (m, 7H, Ar-H), 3.82 (s, 3H, -OCH_3_)
**15**	8.41 (s, 1H, -CH=N),7.83-7.11 (m, 8H, Ar-H), 2.36 (s, 3H, -CH_3_)
**16**	13.19 (s, 1H, OH, exchangeable), 8.94 (s, 1H, -CH=N), 7.63-6.93 (m, 7H, Ar-H), 2.33 (s, 3H, -CH_3_).

**Table 3:** ^13^C-NMR (DMSO-d_6_) ( δ, ppm) of azomethines **5**-**16**.

Compd	^13^C-NMR
**5**	160.0, 154.2, 152.4, 147.3, 146.3, 131.0, 125.5, 125.2, 123.2, 115.9, 112.3, 56.1.
**6**	160.2, 152.4, 152.0, 147.3, 131.1, 129.9, 127.2, 125.5, 122.3, 115.9, 112.3, 56.1.
**7**	163.7, 158.6, 146.4, 135.2, 133.3, 128.5, 128.5, 127.9, 125.2, 123.2, 119.8.
**8**	163.7, 152.4, 135.2, 133.3, 130.0, 128.6, 128.5, 127.9, 127.2, 122.3, 119.9.
**9**	161.1, 160.0, 154.2, 146.4, 132.4, 132.1, 125.2, 123.2, 121.4, 120.5, 117.8.
**10**	160.0, 151.9, 136.6, 135.0, 134.5, 130.5, 128.9, 128.6, 128.3, 127.8, 126.8, 126.3, 115.2.
**11**	160.0, 152.2, 151.9, 147.9, 147.3, 135.0, 131.1, 128.5, 128.3, 127.8, 126.7, 126.3, 125.5, 115.9, 115.1, 112.3, 56.1.
**12**	160.0, 159.1, 144.3, 136.5, 134.5, 130.6, 128.8, 122.1, 115.6, 55.8.
**13**	159.9, 159.1, 152.4, 147.3, 144.3, 131.1, 125.5, 115.9, 115.6, 122.1, 112.3, 56.1, 55.8.
**14**	161.1, 160.1, 159.1, 144.3, 132.4, 132.1, 122.1, 121.4, 120.4, 117.8, 115.5, 55.8.
**15**	160.1, 149.1, 136.9, 136.5, 134.5, 130.6, 130.3, 128.9, 122.2, 21.3.
**16**	161.1, 159.9, 149.1, 136.8, 132.4, 132.1, 130.3, 122.2, 121.4, 120.5, 117.7, 21.3.

**Table 4:** Mass spectra of azomethines **5**-**16**.

Compd.	m/z (% Relative abundance)
**5**	272 (M^+.^, 4.25), 227 (2.34), 139 (4.85), 138 (70.25), 107 (76.60), 92 (48.80), 91 (6.91), 65 (100.00).
**6**	227 (M^+.^, 98.26), 211 (21.66), 166 (14.45), 154 (7.12), 139 (5.12), 104 (22.42), 77 (100.00)
**7**	252 (M^+.^, 27.28), 251 (M-1⎤^+.^, 83.41), 205 (56.73), 178 (4.88), 149 (4.88), 138 (54.55), 115 (70.16), 92 (48.50), 91 (47.95), 65 (100.00)
**8**	207 (M^+.^, 33.52), 206 (M-1⎤^+.^, 100.00), 178 (3.25), 128 (13.57), 115 (18.72), 77 (90.33).
**9**	242 (M^+.^, 96.65), 212 (13.38), 195 (37.11), 167 (29.72), 151 (5.91), 120 (44.48), 76 (100.00)
**10**	267 (M+2⎤^+.^, 20.42), 266 (M+1⎤^+.^, 20.79), 265 (M^+.^, 60.68), 230 (2.89), 202 (3.48), 154 (23.00), 127 (100.00), 77 (29.47), 51 (9.14).
**11**	277 (M^+.^, 100.00), 261 (7.13), 216 (6.07), 204 (5.90), 178 (3.99), 154 (19.97), 127 (80.53), 101 (11.97), 85 (44.01), 83 (69.57), 77 (27.60).
**12**	247 (M+2⎤^+.^, 26.19), 246 (M+1⎤^+.^, 15.23), 245 (M^+.^, 77.99), 230 (100.00), 201 (10.17), 167 (27.80), 149 (5.64), 139 (10.67), 114 (4.26), 77 (35.29), 63 (45.11).
**13**	257 (M^+.^, 100.00), 242 (85.72), 227 (3.73), 198 (5.24), 170 (15.75), 154 (7.33), 134 (6.16), 115 (7.02), 77 (4.02), 64 (19.33).
**14**	228 (M+1⎤^+.^, 16.62), 227 (M^+.^, 100.00), 212 (71.79), 183 (4.33), 154 (3.40), 128 (5.33), 105 (3.08), 77 (55.04), 65 (38.15).
**15**	231 (M+2⎤^+.^, 30.49), 230 (M+1⎤^+.^, 33.41), 229 (M^+.^, 90.60), 193 (2.89), 165 (4.57), 150 (2.05), 118 (22.99), 91 (100.00), 77 (9.79), 65 (61.60).
**16**	211 (M^+.^, 100.00), 210 (M-1⎤^+.^, 76.87), 167 (6.29), 120 (12.86), 118 (8.68), 91 (80.65), 65 (74.49).

**Table 5:** % Inhibition of azomethines against *L. major* leishmania.

Compound	*L. major ^a^*
**1**	0.77 ± 0.09
**2**	-
**3**	-
**4**	0.65 ± 0.01
**5**	-
**6**	0.73 ±0.16
**7**	-
**8**	-
**9**	0.59 ± 0.07
**10**	0.61 ± 0.27
**11**	-
**12**	0.57 ±0.11
**13**	0.63 ± 0.04
**14**	0.68 ±0.12
**15**	0.66 ±0.18
**16**	0.81 ± 0.03
**17**	0.62 ± 0.08
**18**	-
**Standard Drug IC_50_ (µg/mL± S.D)**	
Amphotericin B	0.56 ± 0.20

^a^ percentage inhibition activity: 100 = (non-significant; 0.95−0.80 = low; 0.79−0.70 = Moderate; 0.69−0.60 = Good; below 0.59-0.56 = Significant activity).

**Table 6:** % inhibition of azomethines against different types of fungi.

Compound	*C. albicans*	*A. flavus*	*M. canis*	*F. solani*	*C. glabrata*
**1**	0	0	0	0	0
**2**	0	0	0	0	0
**3**	0	0	0	0	0
**4**	0	0	20^a^	35	0
**5**	0	0	30	0	0
**6**	0	0	0	0	0
**7**	0	0	0	0	0
**8**	0	0	0	0	0
**9**	0	0	60	40	0
**10**	0	0	0	0	0
**11**	0	0	0	35	0
**12**	0	0	35	0	0
**13**	0	0	0	0	0
**14**	0	0	20	0	0
**15**	0	0	0	0	0
**16**	0	0	0	0	0
**17**	0	40	0	15	0
**18**	0	0	0	20	0
**Std. Drug MIC (mg/mL)**					
Miconazole	110.8	-	98.4	73.25	110.28
Amphotericin B	-	20.0	-	-	-

^a^ percentage inhibition activity: 0-39 = low (non-significant); 40-59 = Moderate; 60-69 = Good; Above 70 = Significant activity.
